# Diffusiophoresis of a Weakly Charged Liquid Metal Droplet

**DOI:** 10.3390/molecules28093905

**Published:** 2023-05-05

**Authors:** Leia Fan, Jason Lin, Annie Yu, Kevin Chang, Jessica Tseng, Judy Su, Amy Chang, Shirley Lu, Eric Lee

**Affiliations:** Department of Chemical Engineering, National Taiwan University, Taipei 10617, Taiwan; f08524096@ntu.edu.tw (L.F.);

**Keywords:** diffusiophoresis, conducting droplet, liquid metal droplet, chemiphoresis, electrophoresis, Debye–Hückel approximation

## Abstract

Diffusiophoresis of a weakly charged liquid metal droplet (LMD) is investigated theoretically, motivated by its potential application in drug delivery. A general analytical formula valid for weakly charged condition is adopted to explore the droplet phoretic behavior. We determined that a liquid metal droplet, which is a special category of the conducting droplet in general, always moves up along the chemical gradient in sole chemiphoresis, contrary to a dielectric droplet where the droplet tends to move down the chemical gradient most of the time. This suggests a therapeutic nanomedicine such as a gallium LMD is inherently superior to a corresponding dielectric liposome droplet in drug delivery in terms of self-guiding to its desired destination. The droplet moving direction can still be manipulated via the polarity dependence; however, there should be an induced diffusion potential present in the electrolyte solution under consideration, which spontaneously generates an extra electrophoresis component. Moreover, the smaller the conducting liquid metal droplet is, the faster it moves in general, which means a smaller LMD nanomedicine is preferred. These findings demonstrate the superior features of an LMD nanomedicine in drug delivery.

## 1. Introduction

Diffusiophoresis refers to the spontaneous motion of a colloidal particle in response to a concentration gradient in a solution, especially in an electrolyte solution, as the particle motion there is much faster than in a non-electrolyte solution in general [1,2,3]. Due to the lack of undesirable Joule heating effect, it has been extensively used in drug delivery in particular, as concentration/chemical gradients are abundant in the human body and thus can drive the drug-carrying nanomedicines [4,5]. Among various nanomedicines used in drug delivery, the liposome has been the most popular one, which is a dielectric droplet enclosed by a bi-layer lipid membrane with therapeutic medicals dissolved in the interior fluid. Additionally, in microfluidic and nanofluidic operations [6], diffusiophoresis has been proposed to serve as an alternative driving force replacing the conventional electrophoresis in some specific applications [7,8,9,10,11,12,13,14,15,16]. Furthermore, diffusiophoresis of dielectric droplets has also been used in enhanced oil recovery (EOR) in the petroleum industry to increase the crude oil extraction rate by 20% to 40% [17]. Indeed, diffusiophoresis, especially when it is coupled with liquid droplets, provides a very versatile platform in various practical applications in colloid technology.

In addition to the above-mentioned dielectric droplet [18,19,20,21,22], however, there is a very important separate category of fluid droplets that has received vast research interest recently: the conducting liquid metal droplets (LMDs). Liquid metals can be considered as the softest materials with very high electrical conductivities. Hence, they are often regarded as perfectly conducting [23]. Liquid metals assume a liquid state at or near-room temperature. Among them, mercury has been the most well-known liquid metal in human history. However, due to its high volatility and toxicity, it is not suitable to be used in the human body [24]. Other liquid metals, especially gallium (Ga) and its alloys, have very low vapor pressure and toxicity. Gallium has a melting point of about 30 degrees Celsius. Gallium-based alloys such as EGaIn (75% Ga and 25% In) have a melting point of about 15 degrees Celsius. The alloy galistan (68.5% gallium, 21.5% indium and 10% tin) has a melting point as low as −19 degrees Celsius. Low melting point coupled with low toxicity and low vapor pressure make these gallium or gallium-based LMDs excellent candidates in drug delivery applications in the human body [25]. This is reinforced by the unique therapeutic effect of the Ga^3+^ ions in particular, which can be released by the LMDs containing gallium [26]. Efficient transportation of these LMDs in drug delivery is a challenge to be reckoned with in order to enhance the overall performance of these emerging promising nanomedicines.

A moving liquid metal droplet used to be treated as a rigid solid particle. However, many fundamental phenomena observed in practice are inconsistent with this framework [27]. Solid experimental evidence has been demonstrated for an LMD in various phoretic motions under the influence of an imposed electric field that moves with characteristic fluidic features [27]. As a result, an in-depth investigation of the diffusiophoresis of a conducting LMD is crucial in its various practical applications, especially in drug delivery, where diffusiophoresis has been determined to provide a self-guiding electrokinetic environment to convey the nanomedicines toward the intended locations spontaneously like a cruise missile. This is because specific chemicals are often released from the injured or infected areas needing therapy in the human body, such as the calcium ions in bone crack [4]. Moreover, the concentration of therapeutics in the immediate neighborhood of these areas can be significantly increased as the concentration gradient guiding the motion of nanomedicines is strongest there, which is highly desirable in order to maximize the in vivo efficacy of therapy [28].

Compared with the conventional dielectric droplets such as liposomes, the surface of a conducting LMD is equipotential due to its extremely high conductivity, which means it cannot experience an electric driving force in the tangent direction of the droplet surface [29]. This implies that there is no motion-deterring electric Maxwell traction surrounding the conducting droplet [23]. Moreover, there is no surface tension-induced Marangoni effect for a conducting droplet such as an LMD considered here either, as there is no field gradient tangent to the droplet surface of any kind to induce it [27,30]. This has been experimentally demonstrated as well in a recent research paper regarding the electrophoretic motion of an LMD, among other phoretic motions [27]. Hence, there is no need to consider the impact of interfacial tension in the analysis of LMD phoretic motions. The phoretic motions of a conducting droplet are driven purely by the viscous hydrodynamic forces upon the droplet surface from both the exterior and the interior fluid flows.

There has been a surge of research efforts in the literature dedicated to diffusiophoresis in the last decade. Among them, Shin and his coworkers reported many excellent experimental explorations with valuable outcomes [10,11,12,17,28,31]. Lee and his coworkers, on the other hand, launched a series of theoretical studies on diffusiophoretic motions of dielectric droplets as well as soft particles recently, motivated by the possible applications in drug delivery in particular [32,33,34,35,36]. Very recently, the diffusiophoretic motion of a highly charged conducting droplet was investigated by Lee and his coworkers as well, focusing on the chemiphoresis component in particular, where the droplet motion is induced solely by the solute concentration gradient [30]. Many interesting features were detected there. In particular, it was discovered that a conducting droplet is superior to a dielectric droplet as a nanomedicine in terms of self-guiding itself to the intended area via diffusiophoresis. Here, we conduct a theoretical study on the diffusiophoresis of a conducting liquid metal droplet based on a versatile general analytical formula obtained under Debye–Hückel approximation by Lee and his coworkers recently [37]. We focus on the electrophoresis component in particular here in this study, which results from the internal electric field generated by the induced diffusion potential in the bulk electrolyte solution when the diffusivities of cations and anions are different [38,39]. This provides crucial information on the polarity dependence of the droplet motion as the ion species are abundant in the human body and diffusion potential is known to be present in practice. The preferred polarity of the droplet surface as discussed above thus can be ensured in the fabrication stage, which can often be achieved via surface modifications. The charge condition of a conducting droplet surface is further assumed to be of constant surface charge density; in other words, it remains invariant with any varying electrokinetic environment, such as the electrolyte strength. This is also referred to as ideally polarizable, a condition generally adopted in the theoretical treatment of conducting droplets [23,40]. Note that this is not contradictory to the equipotential characteristic of a conducting droplet mentioned earlier, which simply means the electric potential is uniform on the surface of a conducting droplet at any instant. Certainly, it can be both equipotential and of constant surface charge density; they are two different but compatible concepts. As for the diffusiophoresis of a conducting droplet in general, Lee and his coworkers obtained an analytical formula under the Debye–Hückel approximation valid for a weakly charged dielectric droplet recently [41]. This formula is applicable to a conducting droplet situation as well, whose diffusiophoretic behavior in general has never been investigated before. Later on, a similar approximate analytical approach was reported by Ohshima as well, using a conducting mercury droplet as a specific demonstrating example [42,43]. The predictions there are essentially identical to those using the formula derived by Lee and his coworkers [41], as we shall present.

As mentioned above, we consider the weakly charged liquid metal droplet with constant surface charge density here to be consistent with the perfectly polarizable assumption normally adopted in theoretical treatments of highly conducting liquid droplet [23]. Electrostatically speaking, this means the ions in the suspending electrolyte solution are all “indifferent ions” which can suppress the electric double layer due to their presence in the outer diffuse layer, the so-called double layer suppression effect, but cannot penetrate through the inner compact Stern layer and reach the droplet surface and change the droplet surface charges accordingly [44]. Note that for a droplet with constant surface potential, which is rarely present in reality, the droplet surface charges keep on changing with varying κa values, which makes it difficult to analyze the electrokinetic response of the droplet to the varying electrostatic environment, indicated by the value of κa. This has been demonstrated and discussed in detail in corresponding analyses on the diffusiophoresis of a dielectric droplet, both with or without the presence of the induced diffusion potential recently by Lee and his coworkers [33,35].

The results provided here shed light on the fundamental electrokinetic behavior of a conducting LMD in drug delivery, especially its intrinsic difference from the conventional dielectric liposome droplets. This is crucial to the nanomedicine development in particular. Moreover, the portable nature of the analytical formula here provides highly desirable information on any specific systems of interest, which facilitates the possible need for researchers and practitioners in drug delivery involving nanomedicines of LMDs.

## 2. Theory

**Figure 1:**
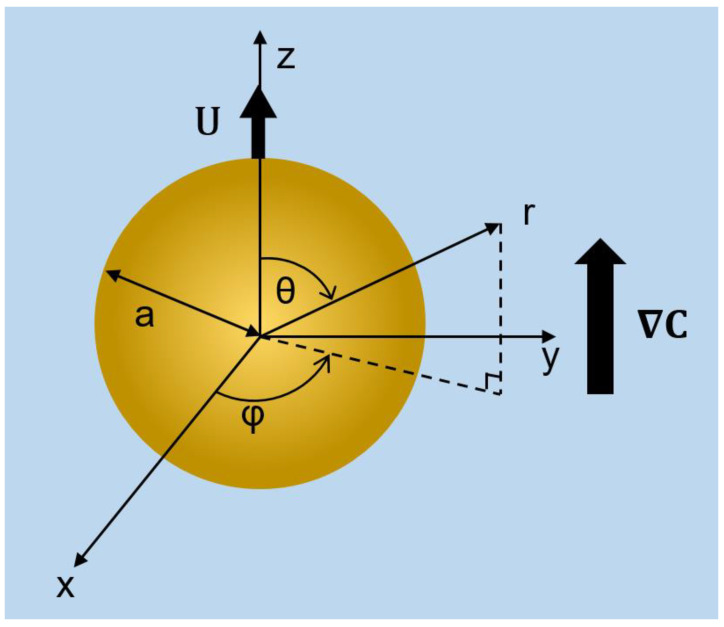
System diagram for a spherical conducting fluid droplet conducting diffusiophoretic motion in symmetric electrolyte solutions.

As shown in Figure 1, the diffusiophoretic motion of a weakly charged conducting liquid metal droplet (LMD) is considered in this study. For simplicity, symmetric binary electrolyte solution systems are further assumed. Extension to non-symmetric binary electrolyte solutions is straightforward. The droplet surface is mobile, ion-impenetrable, and remains spherical without deformation while in the diffusiophoretic motion, justified by the negligible hydrodynamic Weber number [33]. Moreover, as mentioned earlier, the droplet surface is assumed to remain equipotential as well, which is justified for liquid metal droplets due to their extremely high conductivities. All the charges would be uniformly distributed on the droplet surface as well, with no charges present in the interior, as elaborated in the Introduction section earlier.

A concentration gradient ∇C is applied along the Z-direction, which drives the droplet in motion along the same direction as a consequence. Spherical coordinates (r, θ, φ) are adopted with the origin located at the center of the moving droplet.

The governing equations consist of the Poisson equation governing the electric potential, Equation (1), the Nernst–Planck equation governing the ion migrations, Equation (2), the corresponding conservation equation of ion flux as a constraint of possible mechanisms of ion migration, Equation (3), and the momentum equations governing the flow fields for fluids both exterior and interior to the droplet, Equation (4a) and Equation (4b), respectively, which are subject to the incompressibility constraints, Equation (5a) and Equation (5b), respectively [44]:

(1) $$\nabla^{2}\phi =-\frac{\rho }{\varepsilon_{m}}\to a\leq r<\infty ,$$

(2) $$f_{j}=-D_{j}\left(\nabla n_{j}+\frac{z_{j}e}{k_{B}T}n_{j}\nabla \phi \right)+n_{j}v\to a\leq r<\infty ,$$

(3) $$\nabla \cdot f_{j}=0\to a\leq r<\infty ,$$

(4a) $$\eta_{m}\nabla^{2}v-\nabla P-\rho \nabla \phi =0,\to a\leq r<\infty ,$$

(4b) $$\eta_{D}\nabla^{2}v_{I}-\nabla P_{I}=0\to 0\leq r<a,$$

(5a) ∇⋅v=0→a≤r<∞,

(5b) $$\nabla \cdot v_{I}=0\to 0\leq r<a.$$

Note that Equation (1) is the Gauss divergence theorem in electrostatics, where ϕ is the electric potential and ρ the space charge density, with $\rho =\overset{2}{\underset{j=1}{\sum }}z_{j}{\mathrm{en}}_{j}$ for a binary electrolyte solution and n_j_ denoting the number density of ionic species j. Moreover, $\varepsilon_{m\;}$ is the electric permittivity of the ambient electrolyte solution. The first term in Equation (2) stands for the diffusion mechanism of the ion migration, the second term is the electro-migration due to the electrostatic Coulomb force, and the third term is the convective migration, with $f_{j}$ denoting the ion flux of ionic species j. Equation (4a,b) are hydrodynamic Stokes equations for the fluid velocity of the exterior electrolyte solution, v, and velocity of interior liquid metal, $v_{I}$, valid for creeping flow regime, with P as the hydrodynamic pressure. Suffix I indicates the physical variables in the interior of the LMD in general. An extra electric body force term, −ρ∇ϕ, appears in Equation (4a) due to the presence of ions in the exterior electrolyte solution. Additionally, $\eta_{m}$ and $\eta_{D}$ are the viscosities of the ambient electrolyte solution and interior liquid metal, respectively. The details of the definitions of symbols can be found in the List of Symbols.

For a weakly charged droplet, Equation (1) can be replaced by a linear Helmholtz equation. Following the approach by O’Brien and White [45], a linear perturbation analysis is further adopted assuming that the system is only slightly perturbed. The problem is then converted to the one-dimensional dimensionless form in terms of the dimensionless radial distance r*. The resultant governing equations are shown below. The mathematical details can be found elsewhere [46].

(6) $$\nabla^{*}{}_{r}^{2}\phi_{e}^{*}={\left(\kappa a\right)}^{2}\sinh\phi_{e}^{*},$$

(7) $$\nabla^{*}{}_{1D}^{2}{\delta \Phi }^{*}-\frac{{\left(\kappa a\right)}^{2}}{2}\left(n_{10}^{*}+n_{20}^{*}\right){\delta \Phi }^{*}=\frac{{\left(\kappa a\right)}^{2}}{2}\left(n_{10}^{*}G_{1}^{*}+n_{20}^{*}G_{2}^{*}\right),$$

(8) $$\nabla^{*}{}_{1D}^{2}G_{1}^{*}-\frac{d\phi_{e}^{*}}{{\mathrm{dr}}^{*}}\frac{{\mathrm{dG}}_{1}^{*}}{{\mathrm{dr}}^{*}}=-\frac{2{\mathrm{Pe}}_{1}}{r^{*}{}^{2}}\frac{d\phi_{e}^{*}}{{\mathrm{dr}}^{*}}\Psi^{*},$$

(9) $$\nabla^{*}{}_{1D}^{2}G_{2}^{*}-\frac{d\phi_{e}^{*}}{{\mathrm{dr}}^{*}}\frac{{\mathrm{dG}}_{2}^{*}}{{\mathrm{dr}}^{*}}=-\frac{2{\mathrm{Pe}}_{2}}{r^{*}{}^{2}}\frac{d\phi_{e}^{*}}{{\mathrm{dr}}^{*}}\Psi^{*},$$

(10) $$E^{*}{}_{1D}^{4}\Psi^{*}=-\frac{{\left(\kappa a\right)}^{2}}{2}\frac{d\phi_{e}^{*}}{{\mathrm{dr}}^{*}}\left(n_{10}^{*}G_{1}^{*}+n_{20}^{*}G_{2}^{*}\right),$$

where the operators are defined as below:

(11) $$\nabla_{r}^{*}{}^{2}f\equiv \frac{d^{2}f}{{\mathrm{dr}}^{*}{}^{2}}+\frac{2}{r^{*}}\frac{\mathrm{df}}{{\mathrm{dr}}^{*}}=\frac{1}{r^{*}{}^{2}}\frac{\mathrm{df}}{{\mathrm{dr}}^{*}}\left(r^{*}{}^{2}\frac{\mathrm{df}}{{\mathrm{dr}}^{*}}\right),$$

(12) $$\nabla_{1D}^{*}{}^{2}f\equiv \frac{d^{2}f}{{\mathrm{dr}}^{*}{}^{2}}+\frac{2}{r^{*}}\frac{\mathrm{df}}{{\mathrm{dr}}^{*}}-\frac{2}{r^{*}{}^{2}}f=\frac{d}{{\mathrm{dr}}^{*}}\left[\frac{1}{r^{*}{}^{2}}\frac{d}{{\mathrm{dr}}^{*}}\left(r^{*}{}^{2}f\right)\right],$$

(13) $$E_{1D}^{*}{}^{2}f\equiv \frac{d^{2}f}{{\mathrm{dr}}^{*}{}^{2}}-\frac{2}{r^{*}{}^{2}}f=\frac{1}{r^{*}{}^{2}}\frac{d}{{\mathrm{dr}}^{*}}\left[r^{*}{}^{4}\frac{d}{{\mathrm{dr}}^{*}}\left(\frac{1}{r^{*}{}^{2}}f\right)\right],$$

(14) $$E_{1D}^{*}{}^{4}f\equiv E_{1D}^{*}{}^{2}\left(E_{1D}^{*}{}^{2}f\right)=\frac{d^{4}f}{{\mathrm{dr}}^{*}{}^{4}}-\frac{4}{r^{*}{}^{2}}\frac{d^{2}f}{{\mathrm{dr}}^{*}{}^{2}}+\frac{8}{r^{*}{}^{3}}\frac{\mathrm{df}}{{\mathrm{dr}}^{*}}-\frac{8}{r^{*}{}^{4}}f,$$

where ϕ is the electric potential, a is the droplet radius, κa is the dimensionless reciprocal of Debye length indicating the thickness of the double layer. The larger the value of κa, the thinner the electric double layer surrounding the droplet. Moreover, ψ is the stream function representing the flow field of an axisymmetric system whose precise mathematical definition can be found in the List of Symbols. Suffix E refers to the equilibrium state, and the superscript * refers to the dimensionless quantities. δ refers to the perturbation amount of the variables after it. The definitions of the rest of the symbols are contained in the List of Symbols.

Following similar mathematical treatments adopted elsewhere [42], it can be shown that the dimensionless diffusiophoretic mobility for a conducting droplet in general is as follows:

(15) $$\mu^{*}=\frac{{\left(\kappa a\right)}^{2}e^{\kappa a}\zeta^{*}}{3\left(3+\frac{2}{\eta_{\mathrm{fr}}}\right)}\left\{\beta {\left[\mu_{\mathrm{HS}}^{*}\right]}_{E}+\zeta^{*}e^{\kappa a}{\left[\mu_{\mathrm{HS}}^{*}\right]}_{D}+\frac{\beta }{\eta_{\mathrm{fr}}}{\left[\mu_{\mathrm{DL}}^{*}\right]}_{E}+\frac{\zeta^{*}e^{\kappa a}}{\eta_{\mathrm{fr}}}{\left[\mu_{\mathrm{DL}}^{*}\right]}_{D}\right\},$$

where

(16) $${\left[\mu_{\mathrm{HS}}^{*}\right]}_{E}=6E_{-1}\left(\kappa a\right)-6E_{0}\left(\kappa a\right)+\frac{3}{2}E_{3}\left(\kappa a\right)-\frac{3}{2}E_{5}\left(\kappa a\right),$$

(17) $${\left[\mu_{\mathrm{HS}}^{*}\right]}_{D}=3E_{0}\left(2\kappa a\right)-3E_{1}\left(2\kappa a\right)+\frac{3}{4}E_{4}\left(2\kappa a\right)-\frac{3}{4}E_{6}\left(2\kappa a\right)\;+\left[2W_{3}\left(\kappa a\right)+W_{0}\left(\kappa a\right)-3W_{2}\left(\kappa a\right)\right],$$

(18) $${\left[\mu_{\mathrm{DL}}^{*}\right]}_{E}=6E_{-1}\left(\kappa a\right)-4E_{0}\left(\kappa a\right)+E_{3}\left(\kappa a\right),$$

(19) $${\left[\mu_{\mathrm{DL}}^{*}\right]}_{D}=3E_{0}\left(2\kappa a\right)-2E_{1}\left(2\kappa a\right)+\frac{1}{2}E_{4}\left(2\kappa a\right)+\left[2W_{3}\left(\kappa a\right)-2W_{2}\left(\kappa a\right)\right],$$

(20) $$\mathrm{and}\;E_{n}\left(\kappa a\right)\equiv \int_{1}^{\infty }\frac{e^{-\left(\kappa a\right)x}}{x^{n}}\mathrm{dx},\;n\in \mathrm{integer}.$$

β is a dimensionless index defined as $\frac{D_{1}-D_{2}}{D_{1}+D_{2}}$ for a symmetric electrolyte solution, where $D_{1}$ is the diffusivity of cations and $D_{2}$ anions, which indicates the magnitude of the diffusion potential in the bulk electrolyte solution. β=0 indicates the absence of the diffusion potential, such that only the chemiphoresis component is present. The ratio of the droplet viscosity to the exterior electrolyte solution is denoted as ${\;\eta }_{\mathrm{fr}}$. The detailed definitions of the rest of the symbols can be found in the List of Symbols or elsewhere [32,41]. In short, suffix E stands for the contribution from the electrophoresis component by the induced diffusion potential. The suffix D, on the other hand, indicates the contribution from the chemiphoresis component instead. Additionally, suffix HS stands for the contribution of the particle under consideration as a “hard (rigid) sphere”, whereas suffix DL indicates the extra contribution from a liquid droplet with a mobile surface.

Note that although the above analytical formula is of a closed form, it is written in terms of the surface/zeta potential, $\zeta^{*}$, instead. Based on the two–dimensional Gauss divergence theorem at the droplet surface, however, it can be shown that a simple analytical relationship exists for a weakly charged conducting liquid metal droplet considered here:

(21) $$\sigma^{*}=-{\left[\frac{d\phi_{e}^{*}}{{\mathrm{dr}}^{*}}\right]}_{r^{*}=1}=\left(\kappa a+1\right)\zeta^{*}.$$

## 3. Results and Discussion

**Figure 2:**
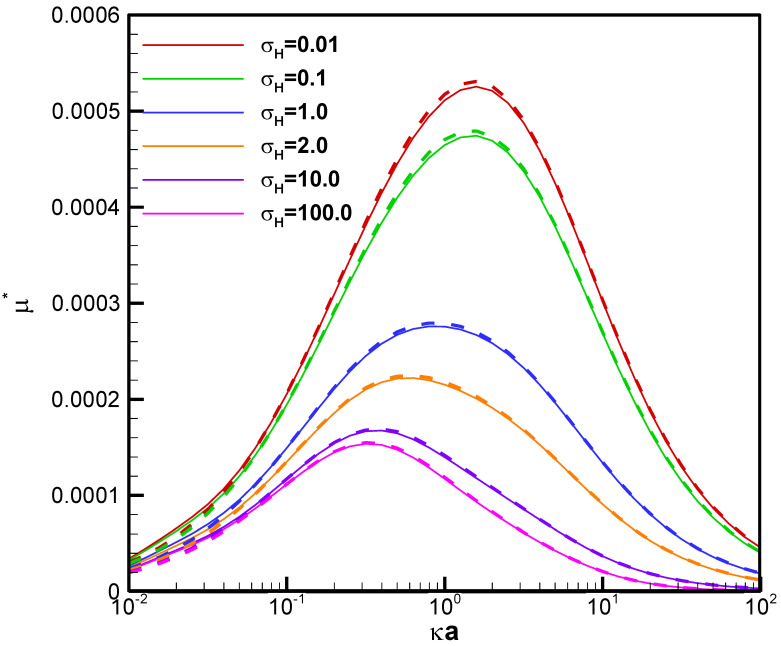
Diffusiophoretic mobility profiles of a conducting fluid droplet with surface charge density σ* = ±0.2 at various viscosity ratios (η_fr_) in KCl solution (β = 0). Dashed lines: Analytical results from Tsai et al. [41]. Solid lines: Results from this study.

We first verify the accuracy of the computation results obtained here based on the general analytical formula [41]. As the analytical formula adopted is derived under Debye–Hückel approximation, which is valid for weakly charged droplets only, we compare its predictions with the numerical results in the literature obtained for the arbitrarily charged conducting droplet in chemiphoresis first to verify its accuracy [30]. As shown in Figure 2, the agreement is excellent for a conducting liquid metal droplet with surface charge density set to 0.2, which corresponds to ϕ_r_ = 0.1 at κa = 1, in an electrolyte solution of KCl (β = 0) with no involvement of diffusion potential. In other words, only the chemiphoresis component is present where the motion of ions is driven solely by the concentration gradient. Additionally, we have also compared with the results available in the literature reported by Ohshima [42] very recently for a weakly charged mercury drop in a KCl solution in particular. Excellent agreement is again observed, which is not shown here for brevity. Note that the droplet system considered there was assumed to be of constant surface potential instead. As a result, some analytical conversion between surface potential and surface charge density was conducted with Equation (21) in the Theory section. Further comparison with the classic results provided by Booth [47] was also made with excellent agreement, which is not shown here for brevity. We thus conclude that the computation results in this study are accurate and reliable and go on to explore the diffusiophoretic behavior of a weakly charged conducting liquid metal droplet based on them.

In general, the unit surface potential, $\phi_{r}$ =1, is regarded as a benchmark weakly charged condition of practical interest in the electrokinetics community. We thus convert the surface charge condition to constant surface charge density via Equation (21) with κa = 1 and $\phi_{r}$ = 1, which yields 2.03 here. This serves as the electrostatic condition of a benchmark weakly charged conducting droplet under investigation here. We explore the chemiphoresis component first, where the droplet motion is induced solely by the concentration gradient of ions in the absence of the diffusion potential. In other words, β = 0, such as in the KCl solution, where the diffusivities of the cations and anions are nearly identical and are often treated as approximately zero [45].

**Figure 3:**
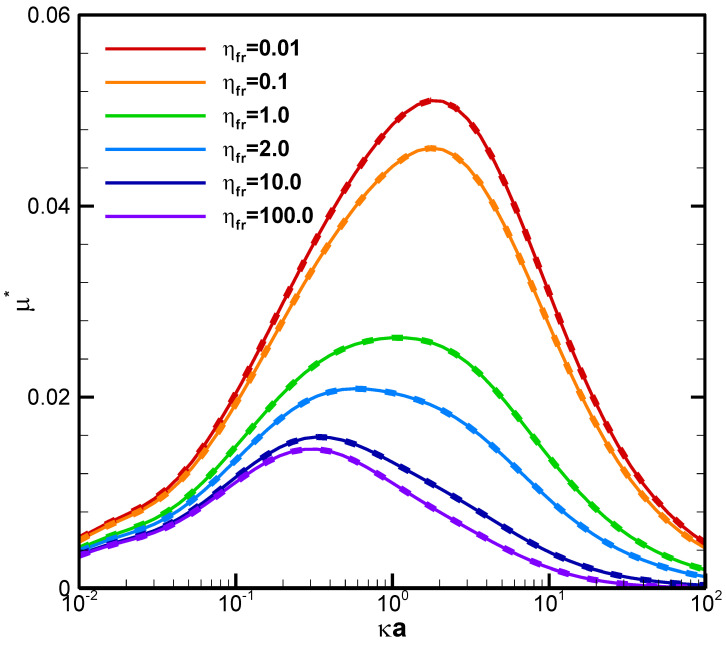
Diffusiophoretic mobility profiles of a conducting fluid droplet with surface charge density σ* = ±2.03 at various viscosity ratios (η_fr_) in KCl solution (β = 0). Solid lines: σ* = +2.03. Dashed lines: σ* = −2.03.

The electric driving force and the retarding hydrodynamic drag force are the two decisive factors to determine the ultimate droplet motion. The electric driving force is directly proportional to the local electric field surrounding the droplet surface and is closely related to the electrostatic environment there, characterized by the electrolyte strength here. The hydrodynamic drag force, on the other hand, is contingent upon the droplet viscosity following the Stokes law. As a result, it would be natural to consider the droplet mobility as a function of the electrolyte strength in the ambient solution for droplet of various viscosities, as shown in Figure 3 for the benchmark weakly charged conducting liquid metal droplets, focusing on the chemiphoresis (β = 0), where κ is the electrolyte strength and a is the droplet radius. Note that κa is a measure of the double layer thickness: The larger the value of κa, the thinner the double layer surrounding the droplet is, as the strong electrolyte strength in the bulk solution suppresses the double layer which is often referred to as the double layer suppression effect [44]. The bell-shape of mobility profiles in Figure 3 results from the fact that the very origin of chemiphoresis is the double layer polarization, which manifests itself at κa around unity. Local maxima are thus observed in Figure 2. At large κa, the surface charges are screened/sheltered by the large amount of counterions adjacent the droplet surface and lead to a significant reduction in effective surface charges. Eventually, the droplet ceases to move, as nearly chargeless condition is reached at very large κa.

It is interesting to note that a conducting liquid metal droplet always moves up the chemical/concentration gradient with positive mobility toward the region of higher concentration of ions, regardless of the droplet viscosity ratios and electrostatic environment, indicated by κa. This is contrary to the corresponding behavior of a dielectric droplet, where negative mobilities are observed most of the time, except at small values of κa, in other words, either weak electrolyte strength or small droplet radius. This implies that a dielectric droplet tends to move down the chemical/concentration gradient of ions to the region with fewer ions [32]. The underlying electrokinetic mechanism is apparently the presence or absence of the electric Maxwell stress, the electrokinetic distinction between a dielectric droplet and an LMD conducting droplet. This fundamental difference in droplet moving directions is critically important in some practical applications of droplets such as in drug delivery. For instance, a nanomedicine made of a conducting droplet (a liquid metal droplet) is superior to a dielectric droplet (a liposome) in terms of self-guiding itself to the desired destination of the injured or infected region in the human body, which often releases specific chemicals to form a strong chemical gradient in its immediate neighborhood [4]. The electrokinetic reason for this tendency of droplets moving up the chemical gradient is very simple: without the help of the Maxwell traction down the chemical/concentration gradient of ions provided by the non-vanishing Maxwell stress tensor upon the droplet surface, the hydrodynamic drag force alone by the downward exterior chemiosmosis flow is simply no match to the intrinsic upward droplet motion induced electrostatically by its surface charges [32]. Moreover, the less viscous a conducting liquid metal droplet is, the faster it moves according to Figure 3, which is contrary to the situation of a dielectric droplet as well, where the viscosity dependence can be reversed under certain electrostatic circumstances due to the dominant tangential electric force upon the droplet surface [32]. For the conducting liquid metal droplet considered here, the stresses exerted upon the droplet surface in the tangential direction are of purely viscous nature as elaborated in the Theory section. Hence, the viscosity dependence of the droplet can be deduced purely from the hydrodynamic consideration, which is the following: The less viscous the droplet is, the faster it moves. Moreover, the smaller a conducting liquid metal droplet is, the faster it moves in general, as shown in Figure 2. This is because the retarding hydrodynamic drag force increases with increasing droplet size in general, according to the famous Stokes law [48].

On the other hand, the droplet moving direction in chemiphoresis is independent of the signs of the charges carried by the droplet. A positively charged droplet moves in the same direction as a negatively charged droplet. In other words, it is the intrinsic nature of the specific droplet under consideration, and there is no way to change it in chemiphoresis. Fortunately, in addition to the chemiphoresis component, there is yet another fundamental element in diffusiophoresis: the electrophoresis component. The electrophoresis component drives the droplet in motion via the electric field generated by the induced diffusion potential in the bulk solution. As mentioned earlier in the Introduction section, diffusion potential is an electric potential generated spontaneously in an electrolyte solution where the diffusivities of cations and anions are different [39,49]. This induced diffusion potential speeds up the slower ions and slows down the faster ions via Coulomb electrostatic force to ensure all the ions migrate at the same speed eventually, which is established instantly in practice [39]. In this way, the electro-neutrality of the system is maintained.

The effect of this induced diffusion potential is exactly like the externally applied electric field in conventional electrophoresis, except that it is simultaneously coupled with the chemiphoresis component in diffusiophoresis. The best merit of it is that it is dependent on the sign of charges carried by the droplet, i.e., its polarity. The impact of the electrophoresis component on a positively charged droplet is significantly different from a that on the negatively charged droplet, and very often drives them to move in opposite directions. This provides a large potential for possible manipulations of droplets by altering their surface charge conditions, both in the fabrication stage and in the operational stage. For instance, the specific electrolyte environment has a profound impact on the surface charge condition of a liquid metal droplet of gallium (Ga), which is an extremely important star nanomedicine in treating some serious diseases [26]. The polarity of the gallium LMD has been determined to be pH-dependent in HCl solution ranging from negatively charged to positively charged in a continuous way [50]. Moreover, it also has been reported as negatively charged in some NaOH solutions [51]. This provides a way to artificially design the polarity of a gallium droplet in its fabrication stage by exposing it to HCl or NaCl solution, for instance. Note that whether there is an electrophoresis component present is contingent upon the specific electrolyte solution under consideration. There must be an induced potential in the electrolyte solution to begin with, such as the NaCl solution; then, the entire strategy of droplet manipulation can possibly work. It is not something that can be added to the system at will.

**Figure 4:**
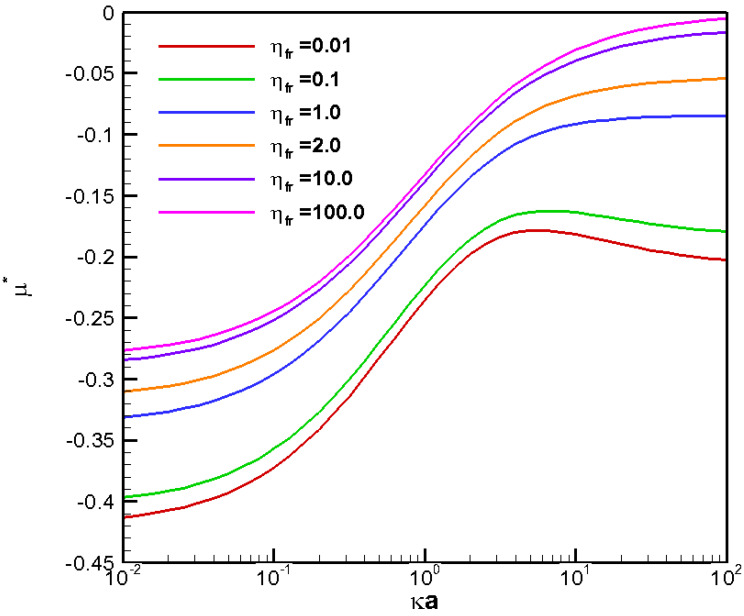
Diffusiophoretic mobility profiles of a conducting fluid droplet with surface charge density σ* = 2.03 at various viscosity ratios (η_fr_) in NaCl solution (β = −0.208).

To further investigate the effect of the electrophoresis component in droplet diffusiophoresis, one has to explore the droplet motion in an electrolyte solution where induced potential is present, as shown in Figure 4, where the corresponding droplet mobility profiles in an NaCl solution is presented. The choice of NaCl is based on the fact that Na^+^ and Cl^−^ are two major ions in the human body as well as in seawater. Indeed, it has served as a benchmark system in the exploration of the electrophoresis component. The magnitude and direction of this diffusion potential in a symmetric electrolyte solution are indicated by a dimensionless β index defined as $\frac{D_{1}-D_{2}}{D_{1}+D_{2}}$, where D_1_ is the diffusivity of the cations, and D_2_ is that of the anions. For the NaCl solution, β is equal to −0.208, which indicates that an induced electric field in the same direction of the chemical gradient is generated to speed up the slower cations and slow down the faster anions simultaneously. As a result, this negative electric field drives a positively charged conducting droplet down the chemical gradient instead, contrary to what happens in chemiphoresis shown in Figure 2. The electrophoresis component turns out to be dominant in determining the ultimate droplet motion here, and reverses the upward droplet motion in chemiphoresis completely. The larger the value of κa is, the slower the droplet moves in general, due to the impact of the upward moving tendency of the droplet by the coupled chemiphoresis component as shown in Figure 2. The overall negative mobilities translate to the undesirable droplet moving direction in drug delivery, as discussed earlier. The highly desirable self-guiding nature of diffusiophoresis would be completely lost. Hence, a positively charged conducting droplet should be avoided in practical applications such as an LMD in drug delivery.

**Figure 5:**
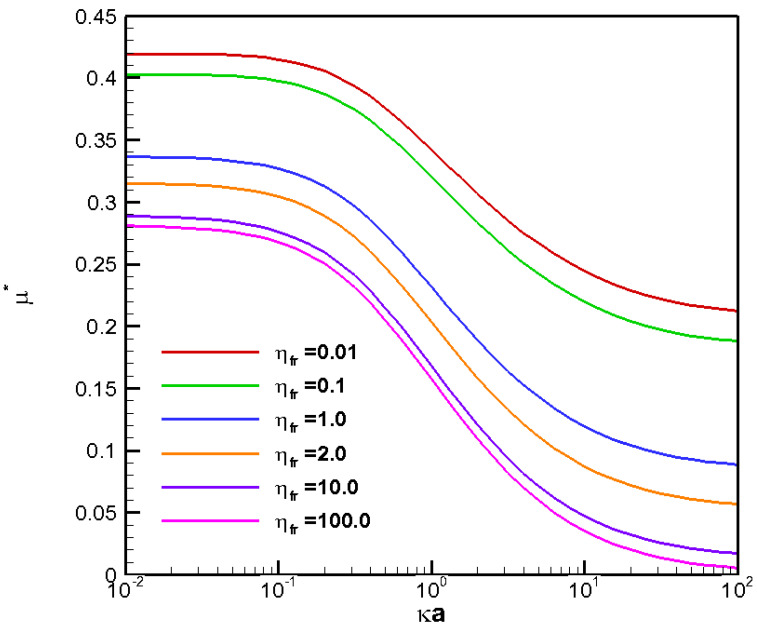
Diffusiophoretic mobility profiles of a conducting fluid droplet with surface charge density σ* = −2.03 at various viscosity ratios (η_fr_) in NaCl solution (β = −0.208).

As mentioned above, polarity dependence of droplet mobility is present in an NaCl solution. Here, we proceed further to explore the droplet motion for a negatively charged droplet instead. The interaction between the electrophoresis component and the diffusiophoresis component is very complicated, which leads to complicated polarity dependences of the droplet motion. As shown in Figure 5, where the corresponding mobility profiles for a negatively charged conducting LMD is depicted, the desired positive mobility up the chemical gradient is observed throughout the κa range investigated. The scenario remains the same regardless of the viscosity ratios. In other words, a negatively charged conducting LMD should always be chosen in drug delivery if the self-guiding nature of diffusiophoresis is of major concern, for instance, the conducting LMD of gallium in the form of $\mathrm{Ga}{\left(\mathrm{OH}\right)}_{4}^{-}$ [27]. As both the electrophoresis and the chemiphoresis components tend to drive the droplet upward along the chemical gradient, the resulting droplet mobility is much larger than the corresponding cases in Figure 3, where only chemiphoresis is present. The dominance of the electrophoresis is very clear. This indicates that the involvement of the electrophoresis component is highly desirable in terms of enhancing the migration speed of the LMD nanomedicine, hence its overall therapeutic performance.

**Figure 6:**
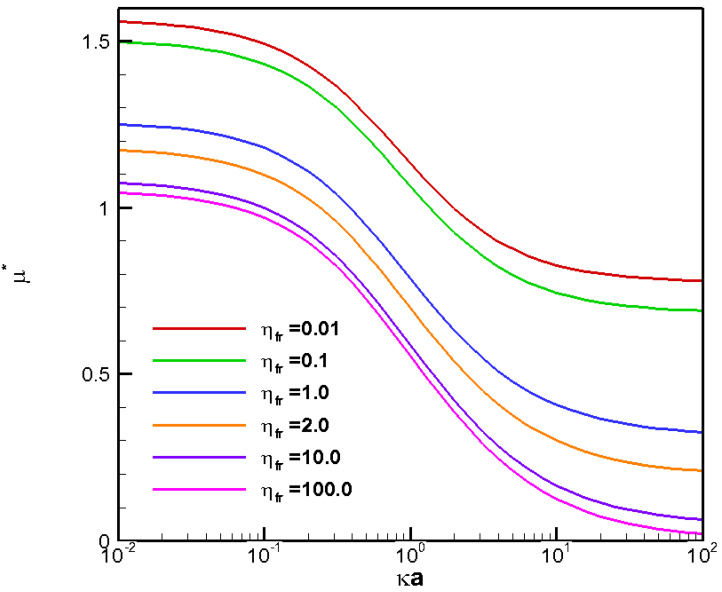
Diffusiophoretic mobility profiles of a conducting fluid droplet with surface charge density σ* = 2.03 at various viscosity ratios (η_fr_) in H_2_CO_3_ solution (β = 0.774).

Moreover, the corresponding droplet mobility profiles in the H_2_CO_3_ electrolyte solution are also examined, as the H_2_CO_3_ solution tends to yield a very large positive diffusion potential (β = 0.774) instead and hence has been proposed to be the driving force in the purification of water [10,12]. The positive β index implies an electric field up along the chemical gradient will be induced to slow down the faster cations and speed up the slower anions in their migration down the chemical gradient. As shown in Figure 6, positive mobilities are observed all the way across the entire range of κa examined for a positively charged conducting LMD as expected, as both components yield positive mobilities in this case.

**Figure 7:**
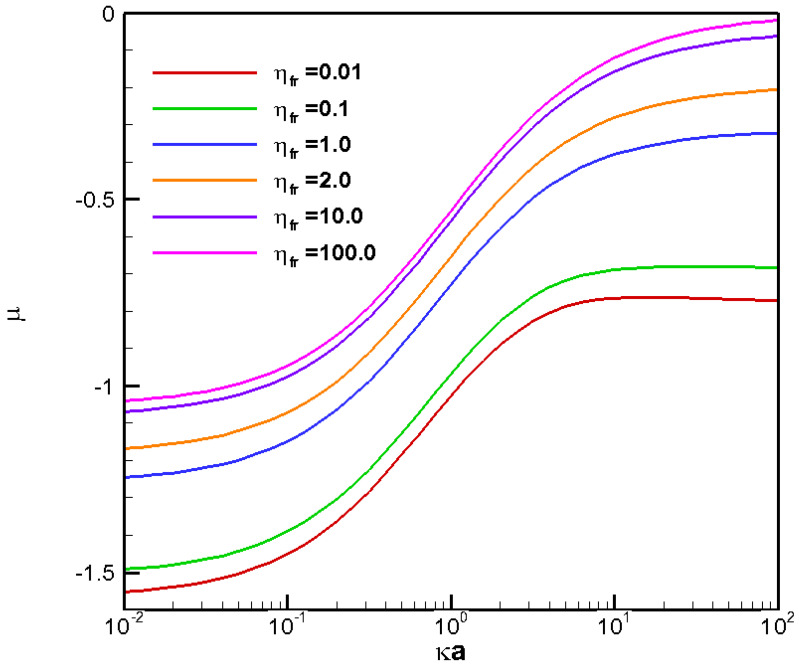
Diffusiophoretic mobility profiles of a conducting fluid droplet with surface charge density σ* = −2.03 at various viscosity ratios (η_fr_) in H_2_CO_3_ solution (β = 0.774).

For completeness, corresponding mobility profiles for a negatively charged droplet in H_2_CO_3_ electrolyte solution are shown in Figure 7 as well. The situation is a bit complicated here due to the duality between the downward moving tendency of the electrophoresis component and the upwardly moving tendency of the chemiphoresis component. However, overall, the droplet still moves downward with negative mobility as the electrophoresis component induced by the large diffusion potential here dominates easily in this case.

## 4. Conclusions

Diffusiophoresis of a weakly charged conducting liquid metal droplet (LMD) is investigated theoretically based on the analytical formula derived under Debye–Hückel approximation. The findings are summarized as follows.

(1) Similar to a highly charged conducting droplet in general, a weakly charged conducting LMD always moves up along the chemical gradient to the region of higher solutes in chemiphoresis, contrary to a dielectric droplet where the droplet tends to move down the chemical gradient most of the time. The presence or absence of the motion-deterring electric Maxwell traction down the chemical gradient is determined to be responsible for this fundamental difference in droplet moving direction. In particular, this means a conducting LMD is inherently superior to a dielectric droplet in drug delivery if self-guiding of the droplet toward the injured or infected locations in the human body by diffusiophoresis is of major concern, as these locations often release specific chemicals in their neighborhood.
(2) The sign of the charges carried by a conducting LMD is crucial in determining its ultimate moving direction in the presence of the diffusion potential via the electrophoresis component. A positively charged LMD tends to move in the opposite direction of a negatively charged one. This provides the maneuverability needed in the highly desirable self-guiding merit in diffusiophoresis. This is made possible by the appropriate choice of the electrolyte solution and the polarity of the droplet surface accordingly, if such a choice is allowed or possible.
(3) With the involvement of the extra electrophoresis component, the migration speed of a conducting LMD is enhanced significantly in general, indicating the dominance of the electrophoresis component over chemiphoresis component here for a conducting LMD.
(4) The less viscous a conducting liquid metal droplet is, the faster it moves in general. Moreover, the smaller an LMD is, the faster it moves. This means a smaller LMD is preferable in drug delivery, as the migration speed of the LMD is enhanced this way.

The findings presented here provide crucial information on the diffusiophoretic behavior of a weakly charged conducting LMD, especially the electrophoresis component, which may be applicable to a highly charged LMD as well in terms of polarity dependence. Further investigation is necessary, though. The results provide guidelines in the fabrication stage of a conducting LMD nanomedicine in particular in terms of optimizing its overall therapeutic performance.

## Data Availability

The data that support the findings of this study are available within the article.

## Abbreviations

**List of Symbols**	
a	radius of the soft particle (m)
C	concentration of total electrolytes (1/m^3^)
D_j_	diffusivity of ionic species j (m^2^/s)
D_1_	diffusivity of cation in binary electrolyte solution (m^2^/s)
D_2_	diffusivity of anion in binary electrolyte solution (m^2^/s)
e	elementary charge of an electron (1.6 × 10^−19^ coul)
F_d_	magnitude of the hydrodynamic drag force exerted on the droplet (Nt)
F_e_	magnitude of the electric force exerted on the droplet (Nt)
f_j_	molar flux of ionic species j (mole/m^2^/s)
$G_{j}^{*}$	one-dimensional shape function representing the extra non-concentric concentration perturbation of ionic species j (volt)
g_j_	shape function representing the extra non-concentric concentration perturbation of ionic species j (volt)
k_B_	Boltzmann constant (1.38 × 10^−23^ joul/K)
n_j_	number density of ionic species j (1/m^3^)
n_10_	bulk concentration of cations (1/m^3^)
n_20_	bulk concentration of anions (1/m^3^)
Pe_j_	Peclet number of ionic species j (Pe_j_ = U_0_a/D_j_) representing the reciprocal of its diffusivity in a dimensionless form
p	pressure (Nt/m^2^)
r	r-coordinate in spherical coordinates (r, θ, φ)
$r^{*}$	dimensionless r-coordinate in spherical coordinates, defined as r/a
U	diffusiophoretic velocity of the droplet under consideration
U_0_	characteristic velocity in diffusiophoretic motion defined as $\frac{\varepsilon }{\eta_{r}a}{\left(\frac{\mathrm{kT}}{z_{1}e}\right)}^{2}$
U*	dimensionless diffusiophoretic velocity of the droplet in [42] defined as $\frac{3\left(\eta_{D}+\eta_{m}\right)}{3\eta_{D}+2\eta_{m}}\beta \phi_{r}$ following the definitions of symbols here
**v**	velocity vector
z_j_	charge valence of ion species j
β	dimensionless diffusivity difference between the cations and the anions in symmetric binary electrolyte solutions defined as $\beta =\frac{D_{1\;}-\;D_{2}}{D_{1}{+D}_{2}}$
δn_j_	perturbation of the number density
δρ	perturbation of the space charge density
${\delta \Phi }^{*}$	one-dimensional perturbation of the electric potential
δϕ	perturbation of the electric potential
ε	electric permittivity (coul/volt/m)
η	viscosity of the fluid (kg/m/s)
η_D_	viscosity of the conducting droplet interior fluid (kg/m/s)
$\eta_{\mathrm{fr}}$	viscosity ratio defined as $\eta_{D}$/$\eta_{m}$
η_m_	viscosity of the ambient solution (kg/m/s)
θ	θ-coordinate in spherical coordinates (r, θ, φ)
κ	Debye length defined as $\left(\sqrt{\sum_{j=1}^{2}n_{j0}{\left(ez_{j}\right)}^{2}/\varepsilon k_{B}T}\right)$
μ	diffusiophoretic mobility of the particle defined as U∇C
μ*	dimensionless diffusiophoretic mobility of the particle defined as μ* = μU0(ϕ0a)
ρ	space charge density (coul/m^3^)
ρ_fix_	uniform charge density in the outer porous layer of the soft particle (coul/m^3^)
σ	surface charge density (coul/m^2^)
σ*	dimensionless surface charge density defined as $\frac{\sigma a}{\varepsilon_{m}\phi_{0}}$
Φ	one-dimensional version of the electric potential distribution (volt)
φ	φ-coordinate in spherical coordinates (r, θ, φ)
$\Psi^{*}$	one-dimensional version of the stream function
ψ	stream function
ϕ	electric potential (volt)
ϕ_0_	thermal potential in a binary electrolyte solution (ϕ_0_ = kT/z_1_e)
$\zeta^{*}$	dimensionless surface potential of the particle (ϕ_r_ = ϕ/ϕ_0_)
**Operators**	
A_n_	$\;\;A_{n}\left(x\right)\equiv \int_{1}^{\infty }\left(\frac{t}{r^{*}{}^{5-n}}+\frac{1}{r^{*}{}^{6-n}}\right)E_{5}\left[\left(x\right)r^{*}\right]e^{-\left(x\right)r^{*}}{\mathrm{dr}}^{*},\;n\in \mathrm{integer}$
C	$\;C\equiv \frac{1}{2}E_{5}\left(\kappa a\right)+E_{-1}\left(\kappa a\right)$
E^2^	operator defined as E^2^ = $\frac{\partial^{2}}{\partial r^{2}}+\frac{\sin\theta }{r^{2}}\frac{\partial }{\partial \theta }\left(\frac{1}{\sin\theta }\frac{\partial }{\partial \theta }\right)$ in spherical coordinates (r, θ, φ)
E^4^	E^4^ = E^2·^E^2^
$E_{1D}^{*}{}^{2}$	one-dimensional E^2^
$E_{1D}^{*}{}^{4}$	$E_{1D}^{*}{}^{2}\cdot E_{1D}^{*}{}^{2}$
$E_{n}$	$E_{n}\left(t\right)\equiv \int_{1}^{\infty }\frac{e^{-\left(t\right)x}}{x^{n}}\mathrm{dx},\;n\in \mathrm{integer}$
N	$\;N\equiv e^{\kappa a}\left[\left(\kappa a\right)E_{-1}\left(2\kappa a\right)+2E_{0}\left(2\kappa a\right)+\frac{2}{\kappa a}E_{1}\left(2\kappa a\right)+\left(\frac{\left(\kappa a\right)}{2}+\frac{1}{{\left(\kappa a\right)}^{2}}\right)E_{2}\left(2\kappa a\right)+\frac{1}{2}E_{3}\left(2\kappa a\right)\right]$
W_n_	$\;\;W_{n}\left(x\right)\equiv -A_{n}\left(x\right)+E_{3-n}\left(2x\right)+\frac{2}{x}E_{4-n}\left(2x\right)+\frac{1}{x^{2}}E_{5-n}\left(2x\right)$ $\;-\left[\frac{1}{2}E_{5}\left(x\right)+E_{-1}\left(x\right)\right]\left[{\mathrm{xE}}_{4-n}\left(x\right)+E_{5-n}\left(x\right)\right],\;n\in \mathrm{integer}$
∇	gradient operator
$\nabla^{2}$	Laplacian operator
∇·	divergence operator
$\nabla_{r}^{*}{}^{2}$	r-directional Laplacian operator
$\nabla_{1D}^{*}{}^{2}$	one-dimensional Laplacian operator
**Superscripts**	
*	dimensionless variable
**Subscripts**	
1	cation
2	anion
D	the contributions from the chemiphoresis component
DL	the contributions from a “Droplet”
E	the contributions from the electrophoresis component
e	equilibrium state
HS	the contributions from a “Hard Sphere”
j	jth ionic species
